# Understanding Selenium and Glutathione as Antiviral Factors in COVID-19: Does the Viral M^pro^ Protease Target Host Selenoproteins and Glutathione Synthesis?

**DOI:** 10.3389/fnut.2020.00143

**Published:** 2020-09-02

**Authors:** Ethan Will Taylor, Wilson Radding

**Affiliations:** Department of Chemistry and Biochemistry, The University of North Carolina at Greensboro, Greensboro, NC, United States

**Keywords:** coronavirus, COVID-19, selenium, glutathione, glutathione peroxidase 1, protease, selenoprotein, thioredoxin reductase

## Abstract

Glutathione peroxidases (GPX), a family of antioxidant selenoenzymes, functionally link selenium and glutathione, which both show correlations with clinical outcomes in COVID-19. Thus, it is highly significant that cytosolic GPX1 has been shown to interact with an inactive C145A mutant of M^pro^, the main cysteine protease of SARS-CoV-2, but not with catalytically active wild-type M^pro^. This seemingly anomalous result is what might be expected if GPX1 is a substrate for the active protease, leading to its fragmentation. We show that the GPX1 active site sequence is substantially similar to a known M^pro^ cleavage site, and is identified as a potential cysteine protease site by the Procleave algorithm. Proteolytic knockdown of GPX1 is highly consistent with previously documented effects of recombinant SARS-CoV M^pro^ in transfected cells, including increased reactive oxygen species and NF-κB activation. Because NF-κB in turn activates many pro-inflammatory cytokines, this mechanism could contribute to increased inflammation and cytokine storms observed in COVID-19. Using web-based protease cleavage site prediction tools, we show that M^pro^ may be targeting not only GPX1, but several other selenoproteins including SELENOF and thioredoxin reductase 1, as well as glutamate-cysteine ligase, the rate-limiting enzyme for glutathione synthesis. This hypothesized proteolytic knockdown of components of both the thioredoxin and glutaredoxin systems is consistent with a viral strategy to inhibit DNA synthesis, to increase the pool of ribonucleotides for RNA synthesis, thereby enhancing virion production. The resulting “collateral damage” of increased oxidative stress and inflammation would be exacerbated by dietary deficiencies of selenium and glutathione precursors.

## Introduction

Several independent studies have now established a significant association between the outcome of COVID-19 and previously documented regional selenium (Se) status in Chinese cities (1), and a similar relationship between serum Se and mortality in a European cohort of COVID-19 patients (2). These observations invite questions about the mechanisms involved, particularly because they fit into a consistent pattern of a role for Se that has been reported over several decades for a variety of RNA viruses (enteroviruses, hantaviruses, and influenza A) and viruses with an RNA stage (HIV-1 and Hepatitis B virus), as reviewed by various authors (3–5).

Most (but not all) of the biological roles of Se, both as selenocysteine in selenoproteins, and as redox-active Se-containing metabolites, involve interactions with cysteine thiols and disulfides in proteins and peptides, and their various oxidized forms. Like Se, the essential antioxidant and free radical scavenger glutathione (GSH), a tripeptide thiol, has also proven to be an important factor in various viral infections, particularly in HIV/AIDS, as reviewed in section 2.1.2. of Taylor (6), and most recently, in COVID-19 (7, 8). Given their intertwined biochemical roles, there are likely to be common factors and mechanisms underlying the therapeutic importance of Se and GSH in viral infections.

## A Confirmed Molecular Interaction Between SARS-CoV-2 Protease and a Human Selenoprotein

Correlations between COVID-19 clinical outcomes and *both* host Se and GSH status provide important context to a related observation emerging from a proteomics-based study of possible cellular targets of SARS-CoV-2 (SCoV2) proteins. Using affinity-purification mass spectrometry, Gordon et al. identified high-confidence protein-protein interactions between 26 of the SCoV2 proteins and human proteins (9). As bait for interacting human proteins, one of the proteins they used was the SCoV2 main viral protease M^pro^, a cysteine protease also known as nonstructural protein 5 (nsp5). The study also included a catalytically inactive C145A M^pro^ mutant (lacking the active site cysteine), which was also used as a bait protein, in order to discriminate false positives that might bind non-specifically to the M^pro^ active site cysteine via disulfide bond formation. One of the interactions they identified involved the cytosolic form of the selenoprotein glutathione peroxidase, GPX1, which bound strongly to the inactive C145A M^pro^ mutant. However, this interaction with GPX1 was *not* observed with the wild type M^pro^. *This is precisely what one might expect to see if GPX1 is a protease substrate*, because its cleavage would produce two fragments that would necessarily have reduced affinity to the enzyme relative to GPX1.

**Figure 1:**
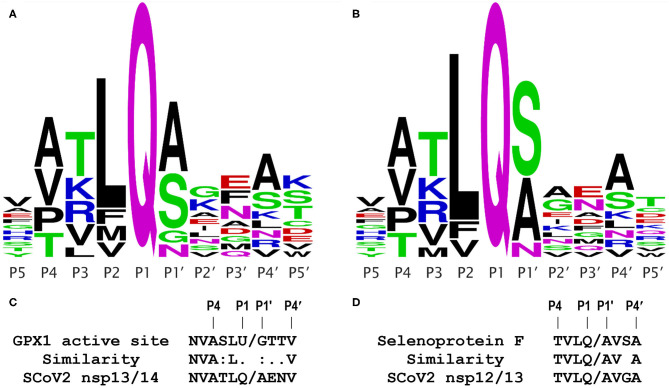
Coronavirus M^pro^ cleavage site consensus sequence logo plots and comparisons. Logo plots from multiple alignments of the known M^pro^ cleavage sites are shown for the 2003 SCoV **(A)** and 2019 SCoV2 **(B)**. The height of a letter at each position reflects its probability in the alignment; each of the logos shown represents the consensus of 11 M^pro^ cleavage sites from a single virus. Plots were generated using WebLogo (weblogo.berkeley.edu/logo.cgi), from the alignments in Figure S1. **(C)** Comparison of the GPX1 active site sequence containing selenocysteine (U) to the known SCoV2 M^pro^ cleavage site at the nsp13/14 junction; this site is identical in the 2003 and 2019 coronaviruses. **(D)** Comparison of a predicted M^pro^ cleavage site in human selenoprotein F to a known SCoV2 M^pro^ cleavage site at the nsp12/13 junction.

Significantly, as shown in Figure 6 in their Extended Data (9), Gordon et al. identified one other host protein (TRMT1) that bound *only* to the inactive M^pro^ C145A mutant, and concluded it was a likely M^pro^ substrate, because they were able to identify a putative M^pro^ cleavage site in the TRMT1 protein sequence (PRLQ/ANFT), where the slash (/) represents the cleavage site. Thus, *the only piece of evidence lacking to draw a similar conclusion for GPX1 is a candidate M*^*pro*^ *cleavage site*, which most algorithms will fail to find, e.g., if they are highly stringent about requiring a Q in the P1 position (Figure 1).

As shown in Figure 1A for the 2003 SARS coronavirus (SARS-CoV, SCoV) and in Figure 1B for the 2019 SCoV2, the consensus logo patterns of 11 M^pro^ cleavage sites in the viral 1ab polyprotein are essentially identical for the 2 viruses. Specifically, 8 of the 11 sites are fully identical over the 10 residues spanning the P5 to P5′ positions, and only two of the 11 cleavage sites have more than a single amino acid difference within the 10 residue span (Figure S1). This means that (as discussed below) *functional studies of the 2003 SCoV protease are highly relevant to that of the 2019 virus*. From Figures 1A,B, the most important residue positions for M^pro^ recognition are, in descending order, P1, P2, P1′, P4, and P3, with the first 3 being the major determinants, with the minimal P2-P1′ consensus being LQ/(S,A,N,G). The downstream sequence past P1′ is highly variable.

**Figure 2:**
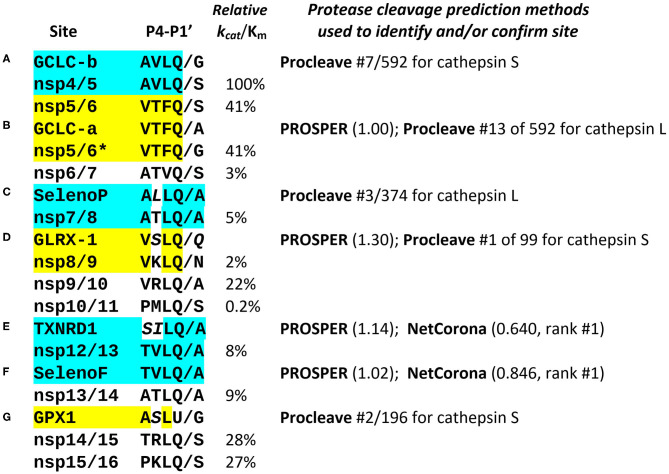
Comparison of known and predicted M^pro^ cleavage sites. The most relevant positions P4-P1′ of the 11 known SARS coronavirus M^pro^ cleavage sites in non-structural proteins are shown (for nsp4 through nsp16), aligned with predicted sites in several human selenoproteins (GPX1, SelenoF, SelenoP, and TXNRD1), as well as glutaredoxin-1 (GLRX-1) and the rate-limiting enzyme for glutathione synthesis, glutamate-cysteine ligase catalytic subunit (GCLC, with 2 predicted cleavage sites **A**, **B**). The known nsp cleavage sites shown are all from SARS-CoV-2, with the addition of the nsp5/6 site from SARS-CoV (marked*). The sites identified in human proteins are displayed next to the known M^pro^ cleavage site to which they are most similar, highlighted in matching color. The experimentally determined catalytic efficiencies of SARS-CoV M^pro^ at each of the known nsp cleavage sites are shown as relative *k*_*cat*_/K_m_ (10). Three different methods for prediction of cysteine protease cleavage sites were used: PROSPER (11), with numbers in parenthesis showing the score for the predicted site, any score >0.8 being significant; NetCorona (12), where scores >0.5 are considered positive, and Procleave (13), where every possible P4-P4′ octamer in a protein sequence is evaluated, scored and ranked. Some aligned non-identical residues highlighted in italics are nonetheless chemically and structurally similar to residues found in the same position in the known cleavage sites, e.g., serine (*S*) vs. threonine (T), Leucine (*L*) or isoleucine (*I*) vs. valine (V), and glutamine (*Q*) vs. asparagine (N); all of these pairs differ only by a single carbon atom (CH_3_ or CH_2_ unit). The predicted M^pro^ cleavage sites in human proteins labeled **(A–G)** are discussed in the text. The computational results cited are collated in Figure S2.

The GPx1 active site has the sequence LUG, where U is the catalytic selenocysteine; this matches the observed M^pro^ consensus core target sequence combination LQ/G in 2 of 3 positions. Furthermore, as shown in Figure 1C, the important upstream side of the known M^pro^ cleavage site at the nsp13/14 junction, NVATLQ/A, is remarkably similar to that of the active site sequence of GPx1, NVASLU/G, where the selenocysteine (U) lines up with a glutamine (Q) in the viral sequence. These two amino acids (U and Q) are not highly similar, but are both midrange in size, and polar in nature, because the selenol residue is predominantly ionized at physiological pH. The other two “mismatches” in the important positions P3 and P1′, i.e., S vs. T and G vs. A, both differ only slightly, by the size of a methyl group, and in any case, having a glycine (G) in the P1′ position is a permitted residue (Figure 1A), as observed in the SCoV M^pro^ cleavage site at the nsp5/6 junction (indicated by ^*^ in Figure 2). Significantly, the Procleave protease cleavage prediction server (procleave.erc.monash.edu.au) (13) identified the GPX1 active site octameric sequence ASLU/GTTV as a highly ranked possible cleavage site for a cathepsin S-like cysteine protease (Figure 2G).

For several reaction intermediates in the GPX1 mechanism, Se is attached to either oxygen, or to nitrogen, as a selenenylamide. The latter is quite stable, and may accumulate during GSH depletion, because GSH is needed to regenerate the selenolate (14). Selenenylamide is more similar than selenocysteine to glutamine, so that form might be preferentially targeted by M^pro^.

Because the mutation rate in cellular genes is thousands of times slower than that for an RNA virus, if there is a physical interaction between these two proteins as Gordon et al. observed using the inactive mutant M^pro^, it is almost certainly because the virus has evolved to target the host for some reason, rather than the converse. The most parsimonious hypothesis is simply that this is a case of a viral protease targeting a cellular gene for cleavage, for which there are abundant precedents, including the proposed M^pro^ targeting of TRMT1 and HDAC2 (9), and HIV-1 protease, which can be toxic to cells, via its action at various cellular targets (15).

## Functional Effects of M^PRO^ in Transfected Cells are Consistent With GPX1 Knockdown

As detailed above, the original 2003 SCoV M^pro^ has essentially identical substrate sequence specificity as that of SCoV2. More specifically, the 10 residue sequence of the cleavage site at the nsp13/14 junction shown in Figure 2A, with the greatest overall similarity to the GPX1 active site sequence, is 100% identical in the 2003 and 2019 SARS coronaviruses (Figure S1). Thus, the results of a 2006 study on the effects of transfecting cells with a SCoV M^pro^ expression vector, in order to study the effects of M^pro^ alone in the absence of intact virus (16), are highly relevant for COVID-19. In transfected human cells, the results were *exactly what one would expect from knockdown of GPX1:* increased oxidative stress via production of reactive oxygen species and activation of transcription factor NF-κB (16). Both of these responses can be produced by exposing cells to hydrogen peroxide, and inhibited by increasing GPX1 activity, either by overexpression or via the addition of sodium selenite to cell culture media (17, 18). Cells expressing the SCoV M^pro^ also became apoptotic, which is a known consequence of NF-κB activation, especially in combination with a high burden of ROS (19), which is even more likely to occur if other antioxidant proteins are also degraded, as discussed below.

## Potential M^PRO^ Sites in Other Selenoproteins and Glutathione-Related Proteins

The question then arises, could targeting of host selenoproteins for proteolysis be part of a more extensive viral strategy contributing to the correlations between Se and GSH status and COVID-19 outcome? To investigate this possibility, we undertook a systematic search for potential M^pro^ cleavage sites in the human selenoproteome and in several GSH-related proteins including glutaredoxin, a small thioredoxin-like CXXC protein that is recycled by GSH, rather than directly by a reductase enzyme. For this analysis, we used three online resources: (1) NetCorona (cbs.dtu.dk/services/NetCorona) (12), the only resource specific for predictions of M^pro^ cleavage sites in coronaviruses (although *not* limited to just the SARS type coronaviruses), (2) PROSPER, the Protease Specificity Prediction Server (prosper.erc.monash.edu.au) (11), which was rated in a recent study as one of the most accurate resources for prediction of HIV-1 protease sites (20), and (3) Procleave (cited above), which was only used if either of the first 2 methods failed to identify a site. Both Procleave and PROSPER do not specifically predict coronavirus cysteine protease sites, but make predictions for more generic cysteine proteases (e.g., cathepsins B, K, L, or S). Thus, for both PROSPER and Procleave, the results had to be screened manually for matches to the M^pro^ cleavage site consensus as shown in Figures 1A,B. This search is also predicated on the assumption that the approach of Gordon et al. (9) might fail to identify some protease targets, because their high affinity interactions are mostly limited to an 8–10 residue sequence, and in some cases may be unable to withstand the conditions needed to be isolable and observable by mass spectrometry.

Figure 2 presents the most notable and highly ranked potential M^pro^ sites identified computationally (the order A-G is not significant, as placement in the listing was determined by similarity to the known nsp sites). The most remarkable instance is in selenoprotein F (SELENOF), an ER protein involved in glycoprotein folding quality control. Over the 8 residues spanning P4 to P4′, it has 7 of 8 identical to the M^pro^ site at the nsp12/nsp13 junction, with the only mismatch at the highly variable P3′ position (Figure 1D), giving a near-perfect match to the M^pro^ consensus. This was the highest scored NetCorona hit, also highly scored by PROSPER, as shown in Figure 2F, and is significant because coronavirus assembly begins in the ER with spike protein accumulation on the ER membrane surface.

Equally important is the hit on the selenoprotein thioredoxin reductase 1 (TXNRD1), which also was highly scored by both NetCorona and PROSPER (Figure 2E). Note that in a larger set of 77 human coronaviruses, a serine (S) in P4 is very common (12), and in addition the sequence SI in the TXNRD1 site is isosteric to TV, the aligned residues in the nsp12/13 site, because transfer of a methyl group from threonine to valine would result in serine and isoleucine. Thus, SI should occupy about the same volume as TV in the M^pro^ active site. Functionally, this predicted cleavage site is only 5 residues from the C terminal of TXNRD1, and would result in removal of the C-terminal redox center of TXNRD1 (AGCUG), making the enzyme incapable of regenerating reduced thioredoxin.

A predicted site in selenoprotein P (SELENOP, Figure 2C) is at position 56, 3 residues prior to the first selenocysteine, such that cleavage here would likely interfere with the N-terminal redox activity, possibly without affecting the Se-transport function of the rest of the protein.

Two possible sites were identified in the catalytic subunit of glutamate-cysteine ligase (GCLC), the rate limiting enzyme for GSH synthesis. The first of these, GCLC-a (Figure 2B), would cleave after position 217, and is an exact match to P4-P1 of nsp5/6, but with an A in P1′, a highly favorable residue for that position, although SCoV has a G there, and SCoV2 has an S. Site GCLC-b (Figure 2A), at position 443, is an exact match to P4-P1 of nsp4/5, but with a G in P1′. The two coronavirus sites with exact P4-P1 matches to GCLC are those at either end of nsp5, i.e., M^pro^ itself, which have the highest catalytic efficiency for cleavage of any of the viral M^pro^ targets (100 and 41%). This suggests that M^pro^ might be particularly efficient at disrupting GSH synthesis, as compared to action at some of the other target sites predicted here, thereby contributing to the clinical findings in COVID-19 (7).

Finally (Figure 2D), we include a non-canonical hit that would cleave at position 90 of glutaredoxin-1 (GLRX-1). This site was very highly scored by PROSPER, as well as Procleave, despite the glutamine (Q) at P1′, which has an additional carbon atom relative to the asparagine (N) found at P1′ in the nsp8/9 site, to which it is otherwise most similar.

## Why Would SARS-CoV-2 Target Components of the Thioredoxin and Glutaredoxin Systems?

Along with GSH reductase, GSH and glutaredoxin comprise the glutaredoxin system, one of two cellular redox systems involved in maintaining reduced thiol states, protein folding and repair, and providing electrons to ribonucleotide reductase (RNR). The other redox system with similar roles is the thioredoxin system, comprised of thioredoxin and thioredoxin reductase (21).

Because DNA is an “add-on” to RNA biosynthesis, deoxyribonucleotides can *only* be synthesized via the reduction of ribonucleotides by RNR, a process which is unsustainable without the participation of one or both of the thioredoxin and glutaredoxin systems. As a consequence of this basic fact of biochemistry, one should expect that RNA viruses might exploit various mechanisms to interfere with components of the thioredoxin and glutaredoxin systems, in order to minimize the diversion of ribonucleotides into DNA synthesis. This is simply the inverse of a strategy used by some large DNA viruses to maximize DNA production, by encoding their own thioredoxin-like proteins, glutaredoxins and even entire RNR genes (22). Consistent with this hypothesis, the results presented here suggest coronaviral targeting of TXNRD1, glutaredoxin-1, and GCLC, a key enzyme for GSH synthesis, for proteolytic cleavage. This would lead to knockdown of *both* of the essential redox systems required to sustain DNA synthesis, and thereby conserve ribonucleotides to enhance RNA synthesis for virus production.

## The Potential M^PRO^ Targets TXNRD1 and GCLC are Strongly Upregulated by Vitamin D3

In light of accruing evidence that vitamin D3 status is inversely correlated with severity of COVID-19 (23–26), it is significant that, via its hormone-like actions on gene expression, D3 has been shown to be a potent activator of both TXNRD1 and GCLC. In a microarray study, 6 h after exposure to 1,25-dihydroxyvitamin D3, TXNRD1 mRNA was upregulated 7.1X, and GCLC was upregulated 2.5X (27). These effects of vitamin D3, as well as a resulting increase in GSH levels, have been functionally documented in cell culture studies (28, 29) and in human subjects (30). *Vitamin D3-mediated activation of both TXNRD1 and GSH biosynthesis could substantially counteract the proteolytic knockdown of TXNRD1 and GCLC predicted by our hypothesis*, which, if validated, would thus represent an important mechanism contributing to a role for vitamin D3 in moderating the severity of COVID-19. However, the therapeutic potential of D3 in COVID-19 is the subject of ongoing debate (31). Perhaps inconsistencies in the findings of various studies of this question may be explained in part by a 20-year old observation, that *the effective upregulation of TXNRD1 by vitamin D3 requires the presence of an adequate level of Se* (28). So the full potential of vitamin D3 vs. COVID-19 may only be seen in combination with optimal Se intake, and possibly, vice-versa.

## Discussion and Conclusions

Altogether, given the protein interaction and functional data (9, 16), it is a very strong hypothesis that GPX1 is an M^pro^ substrate. Of the other sites we propose, some are more likely than others, but those in TXNRD1 and SELENOF are particularly convincing, and all of the predicted sites map to surface accessible regions of the targeted proteins (Figure S3). If validated, these results offer new insights into COVID-19 pathogenesis. If SCoV2 is targeting GSH biosynthesis as well as TXNRD1 and GPx1 for proteolytic knockdown, in infected cells, the resulting decreases in these critical antioxidant molecules would contribute to increased oxidative stress, NF-κB activation and pro-apoptotic signaling (17, 18). Because NF-κB is an activator of many pro-inflammatory cytokines, including IL-6 (32), this could contribute to increased inflammation and the cytokine storms observed in COVID-19, and be a significant basis of pathogenic effects associated with SCoV2 infection of various tissues, including the lung, gastrointestinal tract and cardiovascular system. Importantly, *these consequences of virus-mediated proteolysis would be taking place in everyone who is actively infected, regardless of their Se status*, and would thus be consistent with results suggesting that Se intakes up to several times the minimal dietary requirement are associated with an increase in cure rate from COVID-19 (1). But people with suboptimal nutritional status could be particularly at risk, because their GSH and selenoprotein levels might be low to begin with, making them more vulnerable to the detrimental effects of virus-induced proteolysis. Still, an infection resulting from low dose exposure to virus and limited by a strong immune response in a person with excellent dietary status still might have minimal impact on cellular and patient health, because proteolytic knockdown of host proteins is likely to be incomplete, due to low catalytic efficiencies at some target sites (Figure 2) and the stochastic nature of such molecular interactions.

These results are not unprecedented—our lab has shown that some RNA viruses target host mRNAs encoding isoforms of thioredoxin reductase via RNA:RNA antisense interactions, which, like proteolysis, would likely also result in host selenoprotein knockdown, but by a different mechanism (22, 33). If our hypothesis is confirmed (i.e., some of these host cleavage sites prove to be functional), that leaves us with an interesting question—what evolutionary advantages are driving some viruses to go so far as to degrade or block the synthesis of GSH and specific host selenoproteins?

Understanding why RNA viruses may have developed such strategies presents an interesting challenge for future research.

## Data Availability Statement

All datasets generated for this study are included in the article/Supplementary Material.

## Author Contributions

This study was conceived and designed by ET, who wrote the initial draft. ET and WR worked on the data analysis and revisions of the manuscript. All authors contributed to the article and approved the submitted version.

## Conflict of Interest

The authors declare that the research was conducted in the absence of any commercial or financial relationships that could be construed as a potential conflict of interest.
