# Supplementary material for: Understanding Selenium and Glutathione as Antiviral Factors in COVID-19: Does the Viral Mpro Protease Target Host Selenoproteins and Glutathione Synthesis?
Source: Front Nutr. 2020 Sep 2;7:143. doi: 10.3389/fnut.2020.00143 (PMC7492384; doi:10.3389/fnut.2020.00143)
Supplement: Supplementary file 1 [file Data_Sheet_1.pdf]

Supplementary Materials

for

Taylor & Radding, 2020

M^{pro} site	2003 SARS-CoV	2019 SARS-CoV-2	# Non-identical
nsp4/5	SAVLQ/SGFRK	SAVLQ/SGFRK	0
nsp5/6	GVTFQ/GKFKK	GVTFQ/SAVKR	4
nsp6/7	VATVQ/SKMSD	VATVQ/SKMSD	0
nsp7/8	RATLQ/AIASE	RATLQ/AIASE	0
nsp8/9	AVKLQ/NNELS	AVKLQ/NNELS	0
nsp9/10	TVRLQ/AGNAT	TVRLQ/AGNAT	0
nsp10/11	EPLMQ/SADAS	EPMLQ/SADAQ	3
nsp12/13	HTVLQ/AVGAC	HTVLQ/AVGAC	0
nsp13/14	VATLQ/AENVT	VATLQ/AENVT	0
nsp14/15	FTRLQ/SLENV	FTRLQ/SLENV	0
nsp15/16	YPKLQ/ASQAW	YPKLQ/SSQAW	1

Figure S1. The 11 known M^{pro} cleavage sites in the 2003 and 2019 SARS coronaviruses with a comparison of the 10-residue protein sequences spanning the cleavages sites in the two viruses. The alignments underneath each of the virus names (2003 SARS-CoV and 2019 SARS-CoV-2) were used to generate the logos shown in Fig. 1A and 1B. The cleavage sites and sequences for the SARS-CoV (Genbank NC_004718.3) are given in reference (10), and the equivalent sequences in SARS-CoV-2 were taken from its Genbank Reference Sequence, NC_045512.

S.2 Web server protease cleavage site prediction results cited in Figure 2.

Genbank source files for the protein sequences: **GCLC:** NP_001309424.1; **SELENOP:** NP_005401.3; **GLRX1:** NP_001112362.1; **TXNRD1:** NP_877393.1 **SELENOF:** NP_004252.2; **GPX1:** NP_000572.2

S.2.1. NetCorona results from cbs.dtu.dk/services/NetCorona

NetCorona 1.0 Server - prediction results
Technical University of Denmark

```
   165 SelenoF
MVAMAAGPSGCLVPAFGLRLLLATVLQAVSAFGAEFSSEACRELGFSSNLLCSSCDLLGQFNLLQLDPDCRGCCQEEAQF         80
ETKKLYAGAILEVCGCKLGRFPQVQAFVRSDKPKLFRGLQIKYVRGSDPVLKLLDDNGNIAEELSILKWNTDSVEEFLSE        160
KLERI   Selenocysteine (U) at #96 changed to C for search as NetCorona ignores U
.........................C......................................................         80
................................................................................        160
.....

Pos      Score    Cleavage
_____________________________________
27       0.846    ATVLQ^AVSAF     SelenoF
60       0.067    none     SelenoF
65       0.073    none     SelenoF
75       0.066    none     SelenoF
79       0.064    none     SelenoF
103      0.064    none     SelenoF
105      0.219    none     SelenoF
120      0.136    none     SelenoF
_____________________________________
```

```
   499 TXNRD1
MNGPEDLPKSYDYDLIIIGGGSGGLAAAKEAAQYGKKVMVLDFVTPTPLGTRWGLGGTCVNVGCIPKKLMHQAALLGQAL         80
QDSRNYGWKVEETVKHDWDRMIEAVQNHIGSLNWGYRVALREKKVVYENAYGQFIGPHRIKATNNKGKEKIYSAERFLIA        160
TGERPRYLGIPGDKEYCISSDDLFSLPYCPGKTLVVGASYVALECAGFLAGIGLDVTVMVRSILLRGFDQDMANKIGEHM        240
EEHGIKFIRQFVPIKVEQIEAGTPGRLRVVAQSTNSEEIIEGEYNTVMLAIGRDACTRKIGLETVGVKINEKTGKIPVTD        320
EEQTNVPYIYAIGDILEDKVELTPVAIQAGRLLAQRLYAGSTVKCDYENVPTTVFTPLEYGACGLSEEKAVEKFGEENIE        400
VYHSYFWPLEWTIPSRDNNKCYAKIICNTKDNERVVGFHVLGPNAGEVTQGFAALKCGLTKKQLDSTIGIHPVCAEVFT        480
TLSVTKRSGASILQAGCCG    (Penultimate U changed to C as NetCorona ignores U)
................................................................................         80
................................................................................        160
................................................................................        240
..............................C.................................................        320
................................................................................        400
................................................................................        480
............C.....

Pos      Score    Cleavage
_____________________________________
33       0.068    none     TXNRD1
72       0.104    none     TXNRD1
78       0.071    none     TXNRD1
81       0.123    none     TXNRD1
106      0.073    none     TXNRD1
133      0.071    none     TXNRD1
230      0.075    none     TXNRD1
250      0.070    none     TXNRD1
258      0.065    none     TXNRD1
272      0.505    RVVAQ^STNSE     TXNRD1
323      0.061    none     TXNRD1
348      0.191    none     TXNRD1
355      0.064    none     TXNRD1
450      0.107    none     TXNRD1
464      0.074    none     TXNRD1
494      0.640    ASILQ^AGCCG     TXNRD1
_____________________________________
```

S.2.2. PROSPER, the Protease Specificity Prediction Server, results from prosper.erc.monash.edu.au

S.2.2.B.

B. GCLC-a VTFQ/A **PROSPER** (1.00)
nsp5/6* VTFQ/G

Predicted cleavage sites of individual proteases

Aspartic | Cysteine | Metallo | Serine

cathepsin K | calpain-1

cathepsin K (C01.036)

Position	Segment	Nfragment	Cfragment	Score
322	QLLQEGID	39.19 kDa	33.08 kDa	1.41
206	MGFGMGNC	25.35 kDa	46.92 kDa	1.27
346	TLFEEKIH	42.08 kDa	30.20 kDa	1.08
501	PILNSYLE	60.68 kDa	11.59 kDa	1.07
540	RWMREFIA	65.41 kDa	6.86 kDa	1.07
136	SILEENQA	17.09 kDa	55.19 kDa	1.07
4	MGLLSQGS	0.53 kDa	71.75 kDa	1.05
446	QGMFYFRK	54.01 kDa	18.26 kDa	1.05
361	DHFENIQS	43.85 kDa	28.42 kDa	1.02
217	VTFQACSI	26.69 kDa	45.58 kDa	1.00
242	MALSAASP	29.41 kDa	42.86 kDa	1.00
88	TNPNHPTL	11.01 kDa	61.26 kDa	0.99
295	DSIDSYLS	35.87 kDa	36.40 kDa	0.98

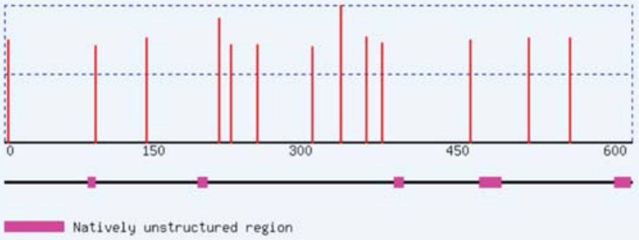

MGLL|SQGSPLSWEETKRHADHVRRHGILQFLHIYHAVKDRHKDVLKWGDEVEYMLVSFDHENKKVRLVLSGEKVLETLQ
EKGERTNPN|HPTLWRPEYGSYMIEGTPGQPYGGTMSEFNTVEANMRKRRKEATSILE|ENQALCTITSFPSTLTRNIRH
RRGEKVVINVPIFKDKNTPSPFIETFTEDDEASRASKPDHIYMDAMGFG|MGNCCLQVTFQ|ACSISEARYLYDQLATIC
PIVMALS|AASPFYRGYVSDIDCRWGVISASVDDRTREERGLEPLKNNNYRISKSRYDSID|SYLSKCGEKYNDIDLTID
KEIYEQLLQ|EGIDHLLAQHVAHLFIRDPLTLFE|EKIHLDDANESDHFE|NIQSTNWQTMRFKPPPPNSDIGWRVEFRP
MEVQLTDFENSAYVVFVVLLTRVILSYKLDFLIPLSKVDENMKVAQKRDAVLQGMF|YFRKDICKGGNAVVDGCGKAQNS
TELAAEEYTLMSIDTIINGKEGVFPGLIPILN|SYLENMEVDVDTRCSILNYLKLIKKRASGELMTVARWMR|EFIANHP
DYKQDSVITDEMNYSLILKCNQIANELCECPELLGSAFRKVKYSGSKTDSSN

S.2.2.D.

D. GLRX-1 V*S*LQ/*Q* **PROSPER** (1.30)
nsp8/9 VKLQ/N

Predicted cleavage sites of individual proteases

Aspartic | Cysteine | Metallo | Serine

cathepsin K

cathepsin K (C01.036)

Position	Segment	Nfragment	Cfragment	Score
90	VSLQQSGE	10.71 kDa	1.96 kDa	1.30
43	QGLLEFVD	5.09 kDa	7.57 kDa	1.20
62	DYLQQLTG	7.33 kDa	5.34 kDa	0.97

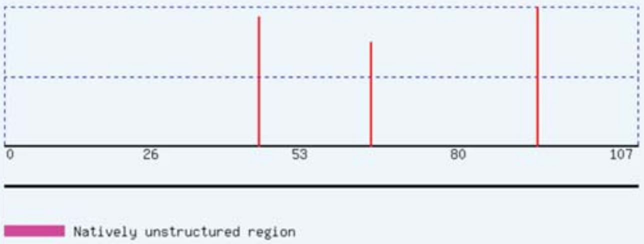

MAQEFVNCKIQPGKVVVFIKPTCPYCRRAQEILSQLPIKQGLL|EFVDITATNHTNEIQDYLQ|QLTGARTVPRVFIGKD
CIGGCSDLVSLQ|QSGELLTRLKQIGALQ

S.2.2.E.

E. TXNRD1 *SI*LQ/A
nsp12/13 TVLQ/A

PROSPER (1.14)

Predicted cleavage sites of individual proteases

Aspartic | Cysteine | Metallo | Serine

cathepsin K | calpain-1

cathepsin K (C01.036)

Position	Segment	Nfragment	Cfragment	Score
494	SILQAGCU	60.06 kDa	0.52 kDa	1.14
406	HSYFWPLE	49.71 kDa	10.86 kDa	1.14
121	VALREKKV	15.05 kDa	45.52 kDa	1.14
184	DDLFSLPY	22.95 kDa	37.62 kDa	1.12
185	DLFSLPYC	23.04 kDa	37.53 kDa	1.12
453	QGFAAALK	55.44 kDa	5.13 kDa	1.12
386	CGLSEEKA	47.17 kDa	13.41 kDa	1.08
348	VAIQAGRL	42.66 kDa	17.92 kDa	1.07
358	QRLYAGST	43.92 kDa	16.66 kDa	0.98
44	LDFVTPTP	5.39 kDa	55.18 kDa	0.95
179	YCISSDDL	22.38 kDa	38.20 kDa	0.95
310	VKINEKTG	38.22 kDa	22.36 kDa	0.94

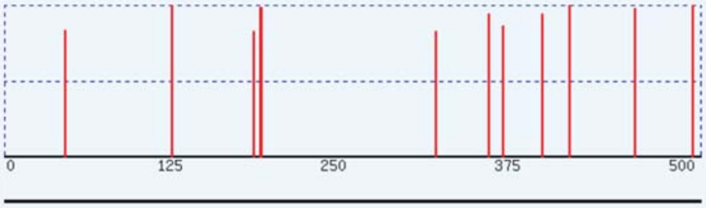

Natively unstructured region

MNGPEDLPKSYDYDLIIIGGGSGGLAAAKEAAQYGKKVMVLDFV|TPTPLGTRWGLGGTCVNVGCIPKKLMHQAALLGQA
LQDSRNYGWKVEETVKHDWDRMIEAVQNHIGSLNWGYRVALR|EKKVVYENAYGQFIGPHRIKATNNKGKEKIYSAERFL
IATGERPRYLGIPGDKEYCIS|SDDLF|S|LPYCPGKTLVVGASYVALECAGFLAGIGLDVTVMVRSILLRGFDQDMANK
IGEHMEEHGIKFIRQFVPIKVEQIEAGTPGRLRVVAQSTNSEEIIEGEYNTVMLAIGRDACTRKIGLETVGVKIN|EKTG
KIPVTDEEQTNVPYIYAIGDILEDKVELTPVAIQ|AGRLLAQRLY|AGSTVKCDYENVPTTVFTPLEYGACGLS|EEKAV
EKFGEENIEVYHSYF|WPLEWTIPSRDNNKCYAKIICNTKDNERVVGFHVLGPNAGEVTQGFA|AALKCGLTKKQLDSTI
GIHPVCAEVFTTLSVTKRSGASILQ|AGCUG

S.2.2.F.

F. SelenoF TVLQ/A
nsp12/13 TVLQ/A

PROSPER (1.02)

Predicted cleavage sites of individual proteases

Aspartic | Cysteine | Metallo | Serine

cathepsin K

cathepsin K (C01.036)

Position	Segment	Nfragment	Cfragment	Score
47	LGFSSNLL	5.30 kDa	14.11 kDa	1.15
155	DSVEEFLS	18.17 kDa	1.24 kDa	1.13
33	SAFGAEFS	3.67 kDa	15.74 kDa	1.04
27	TVLQAVSA	3.02 kDa	16.39 kDa	1.02
30	QAVSAFGA	3.28 kDa	16.13 kDa	1.02
59	DLLGQFNL	6.62 kDa	12.79 kDa	0.99

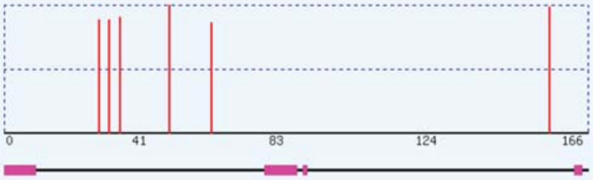

Natively unstructured region

MVAMAAGPSGCLVPAFGLRLLLATVLQ|AVS|AFG|AEFSSEACRELGFS|SNLLCSSCDLLG|QFNLLQLDPDCRGCCQ
EEAQFETKKLYAGAILEVCGUKLGRFPQVQAFVRSDKPKLFRGLQIKYVRGSDPVLKLLDDNGNIAEELSILKWNTDSVE
|EFLSEKLERI

S.2.3. Procleave results from procleave.erc.monash.edu.au

S.2.3.A.

```
A.   GCLC-b       AVLQ/G
     nsp4/5       AVLQ/S
```

ProCleave #7/592 for Cathepsin S

PROTEASE

Cathepsin S (C01.034)

PREDICTED CLEAVAGE SITES

Copy PDF CSV Excel Search:

Rank	Position	P4-P4' Site	N-fragment	C-fragment	Score	Family
1	583	ELLG†SAFR	70.32 kDa	1.95 kDa	0.923	C01.034
2	576	NELC†ECPE	69.46 kDa	2.81 kDa	0.910	C01.034
3	582	PELL†GSAF	70.14 kDa	2.13 kDa	0.832	C01.034
4	5	MGLL†SQGS	0.53 kDa	71.75 kDa	0.803	C01.034
5	243	MALS†AASP	29.41 kDa	42.86 kDa	0.793	C01.034
6	57	YMLV†SFDH	7.10 kDa	65.17 kDa	0.676	C01.034
7	444	AVLQ†GMFY	53.56 kDa	18.71 kDa	0.648	C01.034

S.2.3.B.

```
B.   GCLC-a       VTFQ/A
     nsp5/6*      VTFQ/G
```

ProCleave #13 of 592 for Cathepsin L

PROTEASE

Cathepsin L (C01.032)

PREDICTED CLEAVAGE SITES

Copy PDF CSV Excel Search:

Rank	Position	P4-P4' Site	N-fragment	C-fragment	Score	Family
1	243	MALS†AASP	29.41 kDa	42.86 kDa	0.993	C01.032
2	583	ELLG†SAFR	70.32 kDa	1.95 kDa	0.973	C01.032
3	5	MGLL†SQGS	0.53 kDa	71.75 kDa	0.945	C01.032
4	6	GLLS†QGSP	0.61 kDa	71.66 kDa	0.878	C01.032
5	544	REFI†ANHP	65.80 kDa	6.47 kDa	0.798	C01.032
6	112	QPYG†GTMS	14.09 kDa	58.18 kDa	0.767	C01.032
7	57	YMLV†SFDH	7.10 kDa	65.17 kDa	0.752	C01.032
8	323	QLLQ†EGID	39.19 kDa	33.08 kDa	0.745	C01.032
9	58	MLVS†FDHE	7.19 kDa	65.08 kDa	0.712	C01.032
10	80	ETLQ†EKGE	9.87 kDa	62.40 kDa	0.691	C01.032
11	330	DHLL†AQHV	40.09 kDa	32.18 kDa	0.675	C01.032
12	233	DQLA†TICP	28.38 kDa	43.89 kDa	0.657	C01.032
13	218	VTFQ†ACSI	26.69 kDa	45.58 kDa	0.655	C01.032

S.2.3.C.

C. SelenoP ALLQ/A
nsp7/8 ATLQ/A

ProCleave #3/374 for Cathepsin L

PROTEASE

Cathepsin L (C01.032)

PREDICTED CLEAVAGE SITES

Copy PDF CSV Excel Search:

Rank	Position	P4-P4' Site	N-fragment	C-fragment	Score	Family
1	200	KTVE†TPSP	23.45 kDa	20.25 kDa	0.976	C01.032
2	16	CLLP†SGGT	1.75 kDa	41.96 kDa	0.900	C01.032
→ 3	57	ALLQ†ASUY	6.42 kDa	37.29 kDa	0.892	C01.032

S.2.3.D.

D. GLRX-1 VSLQ/Q
nsp8/9 VKLQ/N

ProCleave #1/99 for Cathepsin S

PROTEASE

Cathepsin S (C01.034)

PREDICTED CLEAVAGE SITES

Copy PDF CSV Excel Search:

Rank	Position	P4-P4' Site	N-fragment	C-fragment	Score	Family
→ 1	91	VSLQ†QSGE	10.71 kDa	1.96 kDa	0.878	C01.034
2	66	QQLT†GART	7.67 kDa	5.00 kDa	0.771	C01.034
3	63	DYLQ†QLTG	7.33 kDa	5.34 kDa	0.660	C01.034
4	48	EFVD†ITAT	5.58 kDa	7.08 kDa	0.550	C01.034
5	89	DLVS†LQQS	10.46 kDa	2.20 kDa	0.465	C01.034

S.2.3.G.

G. GPX1 ASLU/G
nsp13/14 ATLQ/A

ProCleave #2/196 for Cathepsin S

PROTEASE

Cathepsin S (C01.034)

PREDICTED CLEAVAGE SITES

Copy PDF CSV Excel Search:

Rank	Position	P4-P4' Site	N-fragment	C-fragment	Score	Family
1	170	KFLV†GPDG	19.79 kDa	4.08 kDa	0.895	C01.034
→ 2	50	ASLU†GTTV	5.18 kDa	18.69 kDa	0.770	C01.034
3	26	RPLA†GGEP	2.43 kDa	21.44 kDa	0.759	C01.034

Figure S3. Mapping of predicted M^{pro} cleavage sites to accessible surface regions of the target proteins

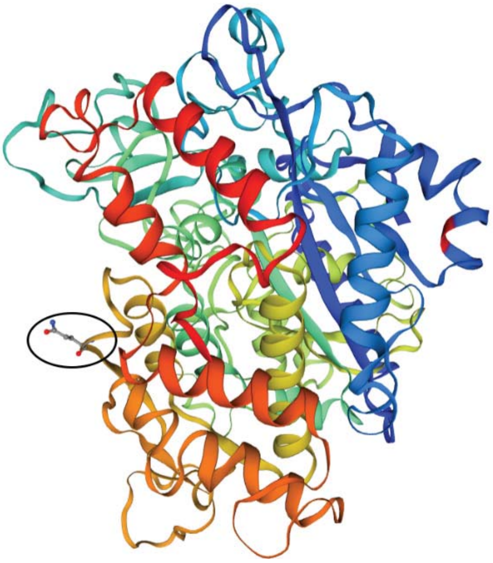

Fig. S3-A. Homology model of GCLC showing location of site GCLC-b, AVLQ/G (circled). The Gln in P1 is shown. The GCLC homology model used in lieu of an X-ray structure is based on the yeast enzyme PDB file 3ig5.1 and is found here: swissmodel.expasy.org/repository/uniprot/P48506

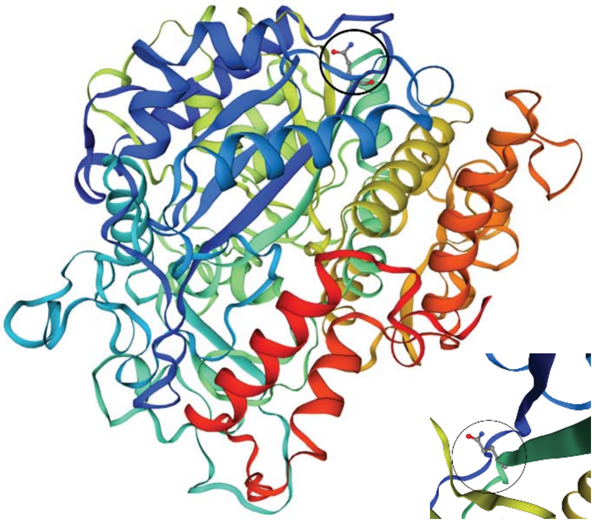

Fig. S3-B. The same homology model of GCLC showing location of site GCLC-a, VTFQ/A (circled). The Gln in P1 is shown. The site is at the protein surface but is recessed in a channel (inset). There is some uncertainty in the 3D structure, but an experimental structure was not available.

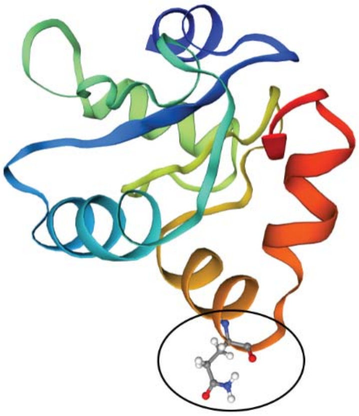

Fig. S3-D. NMR structure of human GLRX-1 (PDB file 1jhb). The predicted M^{pro} cleavage site region shown in Fig. 2D, VSLQ/Q, is circled and the Gln at P1 is displayed.

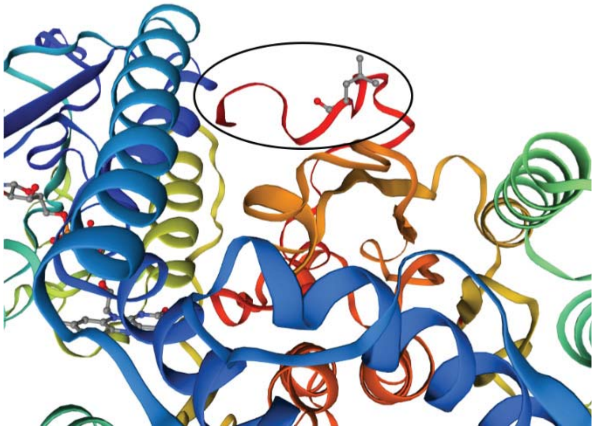

Fig. S3-E. X-ray structure of TXNRD1 (PDB file 2zzc). The C-terminal with the predicted M^{pro} cleavage site region shown in Fig. 2E, SILQ/A, is circled and the Leu at P2 is displayed. The AGCUG C-terminal redox center of TXNRD1 would be removed by M^{pro} cleavage at this site.

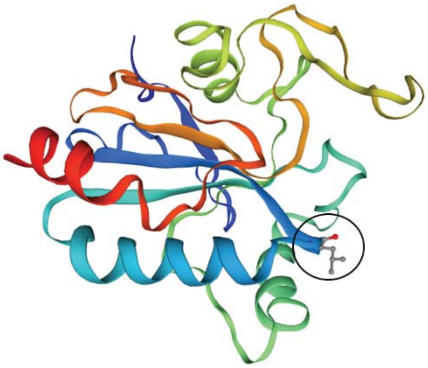

Fig. S3-G. X-ray structure of monomeric GPX1 (PDB file 2f8a). The predicted M^{pro} cleavage site region shown in Fig. 2G, ASLU/G, is circled and the Leu at P2 is displayed.

Fig. S3-C and S3-F: Structures were not available for SELENOF and SELENOP, but for these proteins, the predicted M^{pro} cleavage site was either near the N-terminal ER signal peptide cleavage site (position 27 of SELENOF) or right before a redox center active site (position 56 of SELENOP), so that a high probability of surface accessibility can be inferred in both cases.
